# Measuring Perceived Research Competence of Junior Researchers

**DOI:** 10.3389/fpsyg.2022.834843

**Published:** 2022-04-19

**Authors:** Sarah A. Marrs, Carla Quesada-Pallarès, Korinthia D. Nicolai, Elizabeth A. Severson-Irby, J. Reinaldo Martínez-Fernández

**Affiliations:** ^1^College of Health Professions, Virginia Commonwealth University, Richmond, VA, United States; ^2^Applied Pedagogy Department, Universitat Autònoma de Barcelona, Barcelona, Spain; ^3^Serra Hunter Fellow, Catalonia, Spain; ^4^School of Education, Virginia Commonwealth University, Richmond, VA, United States; ^5^Cognitive, Developmental, and Educational Psychology Department, Universitat Autónoma de Barcelona, Barcelona, Spain

**Keywords:** research competence, doctoral programs, measurement, confirmatory factor analysis (CFA), validity, reliability, perceived competence

## Abstract

Graduates of doctoral (Ph.D.) programs are expected to be competent at designing and conducting research independently. Given the level of research competence needed to successfully conduct research, it is important that assessors of doctoral programs (e.g., faculty and staff) have a reliable and validated tool for measuring and tracking perceived research competence among their students and graduates. A high level of research competence is expected for all Ph.D. graduates worldwide, in addition to in all disciplines/fields. Moreover, graduates of Ph.D. programs may complete their studies in one country but then obtain a research position in another country, emphasizing the need to ensure that all doctoral programs are fostering similar levels of research competence. Thus, the purpose of this study was to gather additional evidence for validity and reliability of the Research Competence (R-Comp) scale. Specifically, we sought to extend the findings of by adapting the scale, translating it to other languages, and applying the tool with a sample of early stage researchers. Our findings provide initial evidence that the adapted PR-Comp is appropriate for use in three languages and across a variety of disciplines/programs of study.

## Measuring Perceived Research Competence of Junior Researchers

The degree doctor of philosophy (Ph.D.) is considered a research intensive degree, designed to foster the development of independent researchers. Upon completion of a Ph.D. program, individuals are expected to be not only experts in their chosen field but competent in designing and conducting research independently following the rules of science (De Jong, 2021); this expectation is shared across the globe (e.g., Hambrick, 1997; Pole, 2000; Trotter, 2003; Jackson, 2013; Pinto et al., 2013; Poh and Kanesan Abdullah, 2019; De Jong, 2021; Fairman et al., 2021; MacLeod and Urquiola, 2021). However, and despite conventional beliefs, doctoral study alone may not be adequately preparing students for performing their roles as independent researchers. Given the level of research competence needed for graduates of doctoral programs – particularly for those who go on to become academic researchers – it is important to have a reliable and validated tool for measuring and tracking perceived research competence among junior researchers (Fairman et al., 2021). Thus, the purpose of this study is to gather evidence for validity and reliability of the Perceived Research Competence (PR-Comp) scale, which could then be used to inform interventions for fostering greater research competence among early stage researchers.

### Research Competence

According to self-determination theory (SDT; Deci and Ryan, 2000), motivation stems from an individuals’ need to satisfy three basic psychological needs, one of which is competence. Competence is thought to satisfy one’s psychological need to master personally challenging tasks (Deci and Ryan, 2000). If the psychological need of competence is met, students may be able to work more effectively and maintain greater well-being; conversely, if competence is not met, students may show signs of negativity (Deci and Ryan, 1985; Sheldon and Elliot, 1999; Andersen, 2000). Perceived competence is one’s perception of both their basic capability of performing a task and a personal judgment of the importance of the task. The importance of perceived competence has also been demonstrated in areas of life such as academic achievement (e.g., Losier and Vallerand, 1994, 1995), work (e.g., Gagné and Deci, 2005; Arshadi, 2010), and sports (e.g., Vansteenkiste et al., 2004; Wilson et al., 2006).

Research specifically focused on perceived research competence is limited both in quantity and in scope. Regarding graduate student and early career researchers’ perceived competence, research often focuses on broader perceived professional competency (Graesser, 2014; Latorre, 2020), clinical competency (Dozier, 2001), or competency utilizing specific assessments (Ingram et al., 2020) instead of competence carrying out research independently. From what we do know, it seems that students who participate in research activities as part of their training report higher levels of research competence, particularly in the areas of data analysis and applying results to practice (Olehnovica et al., 2015). Jung (2018) recently investigated what factors contributed to doctoral students’ perceived research competence. Research-oriented learning environments positively influenced task-oriented (e.g., critical thinking and problem solving) and idea-oriented (e.g., innovation and creativity) research competencies. Notably, Jung (2018) found that participating in manuscript preparation and dissertation writing did not have a strong influence on the students’ perceived research competence. Findings from other studies suggest that the more students are exposed to various aspects of the research process, such designing and carrying out studies, performing literature searches, and publishing manuscripts, the more confident students are in their ability to do research (Phillips and Russell, 1994; Lambie and Vaccaro, 2011; Lambie et al., 2014; Petko et al., 2020). Particularly important for academic researchers, especially those entering the tenure-track, perceived competence in one’s research abilities may also be linked to interest in conducting research, research productivity, innovation, and creativity for both graduate students and early career researchers (Olehnovica et al., 2015; Jung, 2018; Petko et al., 2020). Given the influence perceived competence may have on both academic and non-academic outcomes, coupled with the expectation that graduates of doctoral programs in all fields can effectively conduct research independently, developing a valid and reliable tool for measuring perceived research competence across disciplines and settings is needed.

Recognizing the need for programs to be able to assess the degree to which they were producing competent researchers, Böttcher and Thiel (2018) developed the Research Competence (R-Comp) questionnaire. The R-Comp, a self-report measure of one’s research competence, was designed to measure research competence across multiple disciplines. It was created in alignment with the RMRC-K model, which posits research competence as comprising five dimensions: skills in **R**eviewing the state of research, **M**ethodological skills, skills in **R**eflecting on research findings, **C**ommunication skills, and content **K**nowledge (Thiel and Böttcher, 2014). Their resulting instrument consisted of five factors, one for each dimension of research competence. Though the R-Comp was intended to measure competence across multiple disciplines, it was developed using a sample of students enrolled in a science program at either the Bachelor’s (27.4%), Master’s (68.5%), or doctoral level (4.1%) at a German university. The R-Comp was also developed and administered in German then translated to English only for publication. As such, more work is needed to examine the R-Comp’s utility for measuring research competence among doctoral students and early career researchers broadly. Since the expectations for research competence are similar across fields globally, there is also a need to create a tool that reliably and validly measures perceived research competence across multiple disciplines and languages. As such, the purpose of our study was to gather additional evidence for validity and reliability to support the R-Comp’s intended use of measuring research competence across multiple disciplines. Specifically, and since the original items and factor structure were created and established in German and translated to English for publication purposes only, we sought to replicate Böttcher and Thiel’s (2018) findings by collecting data using the English-translated version of the questionnaire. To begin exploring the appropriateness of using the R-Comp across multiple languages, we also translated the items into two other languages to gather initial evidence for validity and reliability to support broader application of the instrument. Henceforth we refer to the questionnaire as the PR-Comp as we believe this name best captures the intended purpose of the instrument.

## Materials and Methods

### Participants

Current enrollees in or recent graduates of Ph.D. programs were recruited to participate in this study. Participants (*N* = 456) were primarily female (62.7%) and ranged in age from 19 to 64 with the average age being 33.1 (*SD* = 8.09) years of age. Our sample represented 28 nationalities (see Table 1) and 118 disciplines. The vast majority of participants (*n* = 405) were still enrolled in a Ph.D. program; these students’ current year of study ranged from first (21.5%) to five or more (7.9%). Approximately 12% of the sample were recent graduates/early career researchers. Most students (*n* = 365) were enrolled in or completed their doctoral program full-time though 20% were enrolled part-time. Participants reported an average of 5.10 (*SD* = 3.14) years of research experience, including their Ph.D. experience; research experience ranged from not having any experience at all (*n* = 9) to as much as 22 years of research experience (*n* = 1). Lastly, slightly more than half (54.40%) of the sample had not attended any additional education or training on research methods outside of what their Ph.D. program offered.

**TABLE 1:** Nationalities represented by language.

	Frequency by language		
Nationality	Catalan	English	Spanish
Argentinian			2
Belgian		2	
Brazilian	2		2
Bulgarian		1	
Canadian		1	
Chilean	1		17
Chinese		3	1
Colombian		3	8
Costa Rican			1
Ecuadorian	1	1	2
French	1	1	1
German	1	1	1
Ghanian		1	
Greek		2	
Indian		1	
Iranian		4	
Italian		6	
Mexican			115
Paraguayan			4
Peruvian		1	1
South African		1	
South Korean		1	
Spanish	134	4	44
Turkish		1	
United States, American		76	1
Uruguayan	1		1
Venezuelan			2
**Total**	**141**	**111**	**204** *

*The total frequency of those who took the survey in Spanish (i.e., 204) does not equal the total nationalities reported. One respondent did not share their nationality.

### Procedure

We began with a forward and back translation that would result in equivalent versions of the scale in three different languages: Catalan, Spanish, and English. These three languages were chosen as the authors were (1) native speakers of these languages and (2) worked and recruited in countries where these languages are predominantly spoken. The original R-Comp items were translated from German to English solely for publication purposes (see Thiel and Böttcher, 2014). Our process began first by translating the English version of the items into Catalan and Spanish by two native Catalan and Spanish speakers, respectively. Items were translated back into English by a non-native English speaker. Lastly, the items were revised by a native English speaker, at which point revisions and inconsistencies were discussed by the whole group. Part of this process included ensuring that items were unidimensional (i.e., not double-barreled). For example, the R-Comp item “I can confidently apply even complex methods to analyze data/sources/material” was split into the following three items: “I can confidently analyze quantitative data,” “I can confidently analyze qualitative data,” and “I can confidently use a variety of methods for analyzing data (Excel, specialized software, etc.).” Items were also reviewed and edited for clarity when needed. For instance, the item “I am able to plan a research process” was revised to “I am able to plan a research study.” The final list of items were reviewed by native speakers of each language as well as by two individuals who were fluent in each of the three languages. The adapted 36-items of the PR-Comp are presented side-by-side with the original R-Comp items in Table 2. We retained both the 5-point Likert response scale (“Strongly Disagree” to “Strongly Agree”) as well as the five proposed subscales (Böttcher and Thiel, 2018; Böttcher-Oschmann et al., 2021).

**TABLE 2:** The adapted PR-Comp items compared to original R-Comp items by subscale.

RMRC-K model of research competence (Thiel and Böttcher, 2014)	Original R-Comp instrument (Böttcher and Thiel, 2018)	Adapted PR-Comp instrument
**Skills in reviewing the state of research**		
Systematically reviewing the state of research	I know how and where to target a search of the state of research regarding a specific topic.	I know how to conduct a targeted search of the state of research on a specific topic.
		I know where to target a search of the state of research on a specific topic.
	I am able to systematically review the state of research regarding a specific topic.	I am able to systematically review the state of research regarding a specific topic.
Critically evaluating the state of research	Based on the state of research, I am able to identify gaps/unaddressed questions for further research.	Based on the state of research, I am able to identify gaps/unaddressed questions for further research.
	I can evaluate the methodological quality of researched findings well.	I can evaluate the methodological quality of research findings well.
**Methodological skills**		
Systematic planning and preparation of the research process	I find it difficult to formulate specific research questions/hypotheses. *(a)*	I find it difficult to formulate specific research questions/hypotheses. *(a)*
	I am able to decide, which data/sources/materials I need to address my research question.	I am able to decide which data/sources/materials I need to address my research question.
	I am able to plan a research process.	I am able to plan a research study.
	I find it difficult to operationalize each step of the research process. *(a)*	I find it difficult to start/initiate each step of the research process. *(a)*
Selection and application of methods	I find it easy to decide, which methods I need to use to examine a specific research topic.	I find it easy to decide which methods I need to use to address a specific research question.
	I am good at judging which method is inappropriate to answer a specific research question.	I am good at judging which method is inappropriate to answer a specific research question.
	I can apply different research methods appropriate to my research question.	I can apply different research methods appropriate to my research question.
	I can confidently apply even complex methods to analyze data/sources/materials.	I can confidently analyze quantitative data
		I can confidently analyze qualitative data
		I can confidently use a variety of methods for analyzing data (excel, specialized software, etc.)
**Skills in reflecting on research findings**		
Theoretically and methodologically reflecting on results	I am able to adequately interpret my own research findings by relating them to key theories in the subject area.	I am able to adequately interpret my research findings.
		I am able to adequately relate my research findings to key theories in the subject area.
	I am able to critically reflect on methodological limitations of my own research findings.	I am able to critically reflect on methodological limitations of my own research findings.
Reflecting on scientific and practical reach	I am able to reflect on the implications of my own research findings on my discipline.	I am able to reflect on the implications of my own research findings in my discipline.
	I am able to discuss my research findings with regard to their potential applications.	I am able to discuss my research findings with regard to their potential applications.
Reflecting on ethical implications	I am able to critically reflect on the social/ethical implications of my research.	I am able to critically reflect on the social and ethical implications of my research.
	I am able to take a stand on social/ethical issues of research in my discipline.	I am able to take a stand on social and ethical issues of research in my discipline.
**Communication skills**		
Writing academic publications	I can write up research findings in accordance with the current conventions in my discipline.	I can write up research findings in accordance with the current conventions in my discipline.
	I am able to write a publication in accordance with the standards of my discipline.	I am able to write a publication in accordance with the standards of my discipline.
	I find it difficult to write a report that meets the standards of academic writing. *(a)*	I find it difficult to write a report that meets the standards of academic writing. *(a)*
Presentation of research findings	I am able to prepare research findings for a presentation at a research colloquium.	I am able to prepare research findings for a presentation at a research colloquium.
	I am able to present my research at a scientific meeting in accordance with current standards in my discipline.	I am able to present my research at a scientific meeting in accordance with current standards in my discipline.
**Content knowledge**		
Knowledge of central theories and current findings	I have a good overview of the main (current) research findings in my discipline.	I have a good overview of the main (current) research findings in my discipline.
	I am informed about the main (current) theories in my discipline.	I am informed about the main (current) theories in my discipline.
	I am informed about the history of theory/paradigm shifts in my discipline.	I am informed about the history of theory/paradigm shifts in my discipline.
Knowledge of central research methods	I have a sound knowledge of the main research methods in my discipline.	I have a sound knowledge of the main research methods in my discipline.
	I would describe my methodological knowledge as sophisticated and comprehensive.	I would describe my methodological knowledge as sophisticated and comprehensive.
	I am very familiar with different research methods in my subject area.	I am very familiar with different research methods in my subject area.
Knowledge of communication standards in academic research	I am informed about the most important national and international academic publication outlets in my discipline.	I am informed about the most important national and international academic publication outlets in my discipline.
	I am informed about the standards for academic publications that apply in my discipline.	I am informed about the standards for academic publications that apply in my discipline.
	I am informed about the standards that apply to the presentation of research findings at congresses and meetings in my subject area.	I am informed about the standards that apply to the presentation of research findings at conferences and meetings in my subject area.

*(a) reversed items.*

Using an electronic recruitment campaign, a non-probabilistic sample of students and recent graduates from doctoral programs in Mexico, Spain, and the United States were recruited to participate in the study. Students were recruited from programs in these countries since the authors themselves worked in or had connections to programs in these countries. Additionally, students currently and recent graduates of doctoral programs were recruited to participate using social media advertisements. Consenting participants who received the link to the questionnaire either *via* email or by seeing the study advertisement on social media then completed the questionnaire online, on their own time, in one sitting. Participants were informed their participation was completely voluntary and anonymous. After consenting, participants could choose to complete the questionnaire in either Catalan (*n* = 141), English (*n* = 111), or Spanish (*n* = 204). Data collection took place from late fall of 2019 to summer of 2020.

### Data Analysis

Data were screened for response patterns prior to analyses. Descriptive analyses were conducted and both Cronbach’s alpha and McDonald’s omega were calculated to examine the internal consistency of the five subscales in each language. Once internal consistency and normality within each language were established, we then conducted confirmatory factor analysis (CFA) using all data to confirm the five-factor structure of the PR-Comp and provide added evidence for validity based on internal structure. Model fit was assessed using the following indices: comparative fit index (CFI) > 0.95 (Hu and Bentler, 1999); Tucker-Lewis index (TLI) > 0.95 (Hu and Bentler, 1999); root mean square error of approximation (RMSEA) < 0.06 (Hu and Bentler, 1999) and its 90% confidence interval whereby (Kline, 2016). These guidelines and suggestions for “cutoff” points were used to inform overall evaluation of model fit (Marsh et al., 2004). We also compared AIC values of each model, with smaller AICs indicating better model fit (Schermelleh-Engel et al., 2003). The chi-square goodness of fit test was examined to test the null hypothesis that a model fitting the data exactly exists (Kline, 2016). Once we ensured factorial validity of the model, and with the idea of testing invariance across languages, we conducted CFA on all three language-models (Catalan, English, and Spanish), following the recommendations of Sass and Schmitt (2013). Assuming factorial validity of the three language-models was obtained, we then evaluated the configural invariance (as a baseline model) and measurement invariance (both metric and scalar invariance) of the factor model by applying the *forward approach* (sequentially adding more model constraints). To evaluate the model fit, we used Chen’s (2007) cutoff criteria: reject ΔCFI < −0.01 and ΔRMSEA < 0.01. We also considered the need of obtaining non-significant *X ^2^* for the language-models. All data were analyzed using SPSS and AMOS version 23.

## Results

Prior to analyses, we examined descriptive statistics and assessed our data for missingness; our data were complete and as such, there were no missing data patterns identified. However, those completing the PR-Comp in English did tend to have higher scores than those completing the PR-Comp in Catalan and Spanish; all descriptive statistics are provided in Table 3. Subscale scores were normally distributed within each language. Cronbach’s alpha was calculated for each of the five subscales within each language. All alphas were equal to or greater than 0.75 with most being above 0.80, indicating good internal consistency among subscales within each of the three languages (see Table 4). One item on the Communication Skills subscale displayed a low scale-item correlation. Specifically, item 3 showed a low item-scale correlation (0.27); alpha would increase from 0.84 to 0.92 if item 3 was deleted. Three items on the Methodological Skills subscale yielded moderate scale-item correlations; for each of these items, alpha would either increase marginally or not at all if these items were removed. McDonald’s omega was also calculated to ensure the stability of alphas in case one of the assumptions were not met. Omegas tended to be higher than alphas, providing added evidence that subscales produced reliable scores.

**TABLE 3:** Descriptive statistics for PR-Comp subscale.

PR-Comp subscale	Catalan *n* = 141 Mean (*SD*)	English *n* = 111 Mean (*SD*)	Spanish *n* = 204 Mean (*SD*)
Reviewing the state of research	3.53 (*0.87*)	3.85 (*0.89*)	3.64 (*0.86*)
Methodological skills	3.17 (*0.69*)	3.62 (*0.73*)	3.42 (*0.78*)
Skills in reflecting on research findings	3.61 (*0.82*)	3.85 (*0.66*)	3.66 (*0.82*)
Communication skills	3.37 (*0.75*)	3.94 (*0.85*)	3.49 (*0.88*)
Content knowledge	3.35 (*0.87*)	3.65 (*0.68*)	3.43 (*0.85*)

**TABLE 4:** Reliability analyses of the PR-Comp subscales and instrument.

PR-Comp subscales	Catalan	English	Spanish	Combined	Observations
Reviewing the state of research (5 items)	α = 0.88 ω = 0.88	α = 0.93 ω = 0.93	α = 0.92 ω = 0.92	α = 0.91 ω = 0.91	No observations
Methodological skills (10 items)	α = 0.81 ω = 0.84	α = 0.89 ω = 0.90	α = 0.91 ω = 0.91	α = 0.87 ω = 0.89	Items 1, 4, and 9 showed moderate item-scale correlations (0.39,0.44, and 0.42); alpha would increase to 0.88 if item 1 was deleted and remain 0.87 if items 4 and 9 were deleted
Skills in reflecting on research findings (7 items)	α = 0.91 ω = 0.91	α = 0.87 ω = 0.88	α = 0.93 ω = 0.94	α = 0.91 ω = 0.92	No observations
Communication skills (5 items)	α = 0.75 ω = 0.84	α = 0.89 ω = 0.89	α = 0.88 ω = 0.90	α = 0.84 ω = 0.88	Item 3 showed a low item-scale correlation (0.27); alpha would increase to 0.92 if item 3 was deleted
Content knowledge (9 items)	α = 0.93 ω = 0.93	α = 0.91 ω = 0.91	α = 0.94 ω = 0.95	α = 0.93 ω = 0.93	No observations
GLOBAL (36 items)	α = 0.96 ω = 0.97	α = 0.96 ω = 0.96	α = 0.97 ω = 0.97	α = 0.97 ω = 0.97	

*α = Cronbach’s alpha; ω = McDonald’s omega.*

**FIGURE 1:**
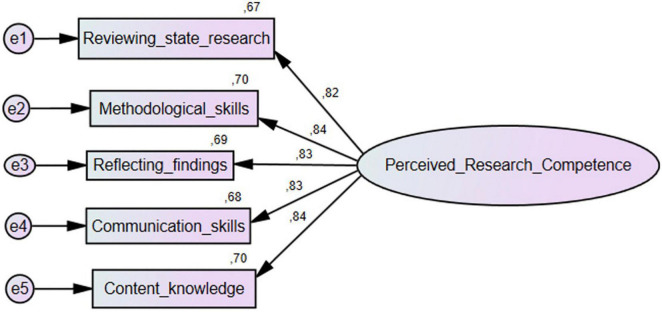
Five-factor model of 36-item PR-comp scale.

**FIGURE 2:**
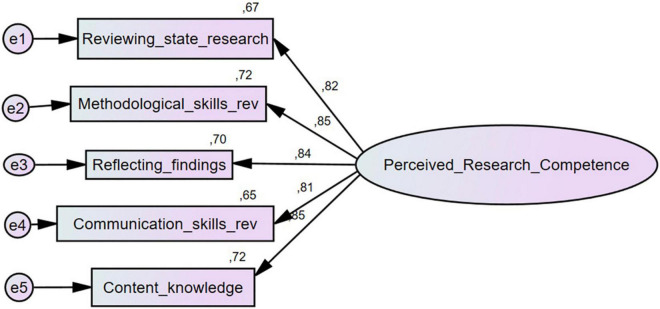
Five-factor model of reduced PR-comp scale.

Confirmatory factor analyses were conducted to examine whether the proposed five-factor structure could be confirmed with our data. According to the chi-square goodness of fit test, we reject the null hypothesis that a model that fits the data exactly exists; *X ^2^*(5) = 12.58, *p* = 0.03. To assess model-data fit, we examined the RMSEA and its 90% confidence interval, the CFI, the TLI, and AICs of a five-factor model including all 36 PR-Comp items and a reduced five-factor with items having low or moderate scale-item correlations removed. Deleting items with low moderate scale-item correlations did not result in improved model fit. The full model resulted in a more favorable RMSEA value and a marginally more favorable CFI and TLI values as well as a lower AIC. However, the confidence intervals for the RMSEA provided weaker evidence of good model fit. Taking all of these indices together, the five-factor model including all 36 items was championed (see Table 5 and Figures 1, 2).

**TABLE 5:** Model-fit indices for PR-Comp full versus reduced model.

Model	*n*	Number of freely estimated parameters	*X*^2^ (df), *p*	Root mean square error of approximation	RMSEA 90% Confidence interval	Comparative fit index	Tucker-lewis index	Akaike information criterion
Full five-factor model	456	15	*X*^2^ (5) = 12.58, *p* = 0.03	0.06	90% CI [0.02–0.10]	0.99	0.99	42.58
Reduced five-factor model	456	15	*X*^2^ (5) = 16.72, *p* = 0.01	0.07	90% CI [0.04–0.11]	0.99	0.99	46.72

Model invariance across languages was first evaluated by conducting factorial validity of the model in each language. As seen in Table 6, model-fit indices were not adequate for any of the language-models (M1–M3). Indeed, Hoelter’s value at 0.05 of significance, indicated that M2 and M3 (English and Spanish, respectively) did not obtain the minimum sample size needed according to the complexity of the model, which should be above 200 (Garson, 2015). Thus, we were not able to guarantee factorial validity as a first step for model invariance. Nonetheless, and with the idea of exploring the results of evaluating measurement invariance, we continued with the analyses as if model-fit indices were accepted. As expected, Table 7 showed that there was configural non-invariance (unconstrained baseline model) as well as metric and structural non-invariance. It can be observed when looking at chi-square significance and the cutoff criteria suggested by Chen (2007) at CFI and RMSEA values. Thus, these results suggested that the data collected was not enough to ensure model invariance among languages.

**TABLE 6:** Model-fit indices for PR-Comp language-models.

Model	*X*^2^ (df), *p*	Root mean square error of approximation	RMSEA 90% Confidence interval	Comparative fix index	Tucker-lewis index	AIC	Hoelter *p* = 0.05
M1. Catalan (*n* = 141)	*X*^2^ (5) = 1.62, *p* = 0.898	0.00	0.00–0.05	1.00	1.01	31.63	952
M2. English (*n* = 111)	*X*^2^ (5) = 6.70, *p* = 0.244	0.06	0.00–0.15	0.99	0.99	36.70	182
M3. Spanish (*n* = 204)	*X*^2^ (5) = 21.33, *p* = 0.001	0.127	0.08–0.184	0.98	0.96	51.33	106

**TABLE 7:** Measurement invariance across PR-Comp language-models.

Model (M)	*X* ^2^	df	*p*	CFI	Root mean square error of approximation	Δ *X*^2a^	Δdf	*p*	ΔComparative fix index^a^	ΔRMSEA^a^
M4. Configural invariance	29.63	15	0.013	0.99	0.05					
M5. Metric invariance	56.28	23	0.000	0.98	0.06	26.65	8	0.013	−0.02	0.01
M6. Structural invariance	105.31	33	0.000	0.95	0.07	49.03	10	0.000	−0.03	0.01

*^a^Indicates comparisons to the previous model, M5 with M4 and M6 with M5.*

## Discussion

Our study aimed to further validate Böttcher and Thiel’s (2018) questionnaire as well as gather evidence for validity and reliability to support the use of this questionnaire across multiple disciplines and languages. Our findings provided added evidence that the PR-Comp can be applied across various disciplines and fields of study in addition to confirming the five-factor structure underlying the items, as proposed by Böttcher and Thiel (2018).

The results of our CFA were consistent with that of Böttcher and Thiel (2018) and our analysis confirmed that a five-factor structure fits the data well. This provided validity evidence based on the internal structure of the scale as well as supported the scale’s theoretical alignment with the RMRC-K model of research competence (Thiel and Böttcher, 2014). Furthermore, our results provided evidence for reliability of the scores produced by the PR-Comp both globally and within each language. All alphas were quite high and would either increase marginally or not at all with the deletion of any items. As previously mentioned, one item, item 3 on the Communication Skills subscale, did appear problematic and would result in a notable increase in alpha if it were removed from the scale. Furthermore, this finding was consistent across languages, suggesting this item should be examined further for clarity and relation to other variables in the scale. McDonald’s omega values were also calculated for each subscale score and tended to be higher than alphas. Taken together, both alphas and omegas suggest internal consistency of the subscale items is quite high. However, there are important limitations to our study that limit the generalizability of our findings.

Our findings do provide some initial evidence for external validity as it appears using the PR-Comp scale may be appropriate for use in and produces reliable scores when applied across countries, languages, and fields of study. However, due to the small sample size within each language group, we are not yet able to definitively demonstrate measurement invariance across languages. Future studies should seek to gather added evidence for external validity of the instrument. Another limitation to the broad applicability of our findings is that they are based on responses to a self-reported assessment of one’s own competence, which may limit the accuracy of responses. Participants may have responded more favorably to make it seem as though they were more competent than their true competence. Findings might be more accurate if scores represented another person’s assessment of an individual’s research competence rather than one’s own judgment of their competence. For instance, doctoral program faculty and/or instructors’ ratings of individual students’ competence on each of the PR-Comp items may provide a more objective rating of competence. Moreover, correlating instructors’ ratings of research competence with students’ ratings of research competence could provide convergent validity evidence of PR-comp scores. As research competence and research self-efficacy are often interchangeably used, future researchers could also consider using scores on a research self-efficacy measure as a means of gathering discriminant validity evidence for PR-Comp scores. This information would be useful to broaden our understanding of the theoretical differences between these two constructs. Though the PR-Comp items were drafted and edited by individuals having a Ph.D., it would be beneficial to further evidence for validity based on test content by having doctoral faculty from various fields review the items. This would ensure that items are relevant to all graduates of doctoral programs. Another limitation to note is that our sample was recruited from doctoral programs and/or countries to which the authors had ties or in which the authors worked. While steps were taken to gather data from a large sample that represented various fields and countries as well as languages spoken, our findings might not represent all early career researchers across the globe. Similarly, the majority of our sample were still enrolled in their doctoral programs, meaning our findings are less generalizable to early career researchers and recent graduates of doctoral programs. Finally, future studies should consider investigating measurement invariance (Davidov et al., 2012) to ensure that we are measuring similar constructs within each language group. While our primary purpose was to expand evidence for validity and reliability of the PR-Comp’s original purpose, more work is needed to be sure the scale captures the same construct across languages. Since the original items were created in German and then translated for publication, including data from a German-speaking sample would also be beneficial.

Having a tool for measuring research competence that is appropriate across settings and languages that also produces reliable scores has implications for both practice and research. Regarding practice, doctoral program faculty and staff could use this tool to measure and track their students’ and graduates’ perceived research competence. For example, PR-Comp scores could be used to identify areas of strength and weakness of a program which could then inform intervention efforts to boost research competence in a more targeted way. A tool that can be completed by students would not only be a more efficient means of collecting this data but would enable programs to track individual and cohort student growth over time. This tool could also be used as a means for assessing readiness for doctoral study since a basic understanding of some research concepts is needed prior to beginning doctoral study. Regarding research, the PR-Comp scale is a tool that appears to produce stable and generalizable findings that apply to the broader population of early career researchers and doctoral students. This could lead us to greater understanding of the global landscape of research competence of graduates of Ph.D. programs and, ultimately, support the global efforts of doctoral programs and their quest to train competent researchers (e.g., Hambrick, 1997; Pole, 2000; Trotter, 2003; Jackson, 2013; Pinto et al., 2013; Poh and Kanesan Abdullah, 2019; Fairman et al., 2021; MacLeod and Urquiola, 2021).

## Data Availability Statement

The raw data supporting the conclusions of this article will be made available by the authors, without undue reservation.

## Ethics Statement

The studies involving human participants were reviewed and approved by Virginia Commonwealth University Institutional Review Board (IRB). The patients/participants provided their written informed consent to participate in this study.

## Author Contributions

CQ-P and SM were the leaders of this project and contributed most to the project by designing the study and taking the lead on manuscript preparation. KN and ES-I contributed to data collection efforts and drafted the initial introduction of the study. JM-F supported data collection and reviewed drafts of the manuscript. All authors contributed to data collection efforts and manuscript preparation.

## Conflict of Interest

The authors declare that the research was conducted in the absence of any commercial or financial relationships that could be construed as a potential conflict of interest.

## Publisher’s Note

All claims expressed in this article are solely those of the authors and do not necessarily represent those of their affiliated organizations, or those of the publisher, the editors and the reviewers. Any product that may be evaluated in this article, or claim that may be made by its manufacturer, is not guaranteed or endorsed by the publisher.
